# TC-PTP nuclear trafficking in keratinocytes

**DOI:** 10.18632/aging.101345

**Published:** 2017-12-12

**Authors:** Mihwa Kim, Liza D. Morales, Dae Joon Kim

**Affiliations:** Department of Biomedical Sciences, School of Medicine, University of Texas Rio Grande Valley, Edinburg, TX 78541, USA

**Keywords:** TC-PTP, UVB, keratinocyte, AKT, nuclear translocation

Nucleocytoplasmic trafficking of macromolecules is an important mechanism that can regulate various cellular processes including cell-cycle regulation, gene expression, DNA repair, ubiquitination and RNA processing. Its dysfunction has been linked to aging, aging-associated neurodegenerative diseases, and cancer [1]. Proteins larger than 40 kDa can be shuttled through the nuclear pore complex bound within the nuclear membrane *via* transport receptors of the β-keryopherin family, which includes at least 20 different known importins and exportins. A short string of amino acids on a protein called the nuclear localization sequence (NLS) is recognized by the members of the importin transport receptors and NLS-containing proteins are targeted for localization in the nucleus. Similarly, nuclear export sequence (NES)-containing proteins are recognized by the members of the exportin transport receptors and targeted for export to the cytoplasm. Nucleocytoplasmic trafficking can be regulated in a number of different ways. Above all, phosphorylation of cargo proteins has emerged as a critical mechanism in the regulation of trafficking between the cytoplasm and nucleus [1, 2].

Phosphotyrosine-based signaling is a major regulatory mechanism in cellular homeostasis. The reversible phosphorylation of proteins at specific tyrosine residues is catalyzed by protein tyrosine kinases (PTKs). PTKs are regulated by endogenous negative feedback mechanisms involving protein tyrosine phosphatases (PTPs), which negatively regulate the rate and duration of phosphotyrosine signaling. Substrates of PTPs include various signaling molecules, such as receptor kinases and transcription factors, in both the nucleus and cytoplasm [3]. Subcellular localization of PTPs determines their accessibility to potential target substrates. Therefore, the regulation of the specific localization and translocation of PTPs is important to understanding their functional roles. For example, Pez/PTPD2/PTP36 is a non-transmembrane tyrosine phosphatase with homology to the FERM (ezrin, 4.1, radizin, moesin) family of proteins which are involved in cell cytoskeletal organization and its subcellular localization is regulated by cell density and serum concentration. Pez is localized in the cytoplasm when vascular endothelial cells are confluent or serum-starved, resulting in quiescence. On the other hand, when cells are cultured at low density or fed with serum, Pez is translocated to the nucleus and can induce cell proliferation [4].

TC-PTP (encoded by *PTPN2*) is one of 17 intracellular, nonreceptor PTPs that is ubiquitously expressed in embryonic and adult tissues. TC-PTP has a critical role in hematopoiesis, immune function, and glucose metabolism. TC-PTP is also involved in the suppression of tumor growth of several types of cancers, including breast cancer and colon cancer, through its negative regulation of the transcription factor STAT3. There are two forms of TC-PTP generated by alternative splicing at the 3′ end of the gene: TC45 (TC-PTPa) and TC48 (TC-PTPb). TC45 (45 kDa) is the major form of TC-PTP in most species including human and mouse. TC45 contains a bipartite nuclear localization signal (NLSI and NLSII), while TC48 (48 kDa) contains a hydrophobic C-terminus and is localized to the endoplasmic reticulum [5]. Interestingly, our previous studies have shown that despite its NLS, TC45 is specifically localized within the cytoplasm of skin keratinocytes and it translocates to the nucleus following UVB irradiation [6].

**Figure 1:**
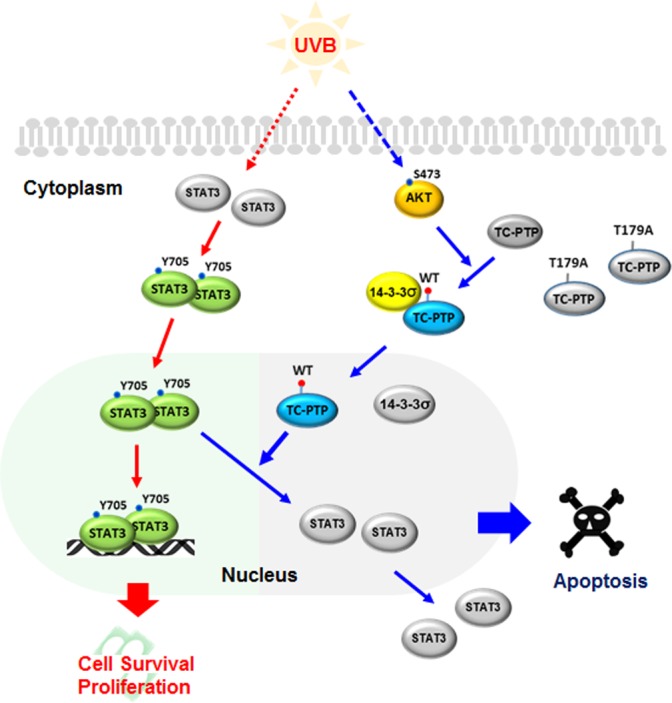
The mechanism of UVB-mediated nuclear translocation of TC45 by the AKT/14-3-3σ axis in keratinocytes UVB-mediated activation of AKT triggers TC45 phosphorylation on T179, which results in the binding of 14-3-3σ with cytoplasmic TC45 and subsequently, their nuclear translocation. In the nucleus, TC45 dephosphorylates STAT3 before it binds to the promoter regions of its target genes, resulting in the inhibition of the genes necessary for keratinocyte survival and proliferation.

In our current study, we elucidate the molecular mechanism by which UVB radiation induces nuclear translocation of TC45 and its significant impact on keratinocyte survival and proliferation. We found that following UVB irradiation of keratinocytes, AKT becomes phosphorylated, resulting in the induction of both 14-3-3σ and TC45 nuclear translocation [7]. We identified several putative 14-3-3σ binding sites within TC45 and substitution mutation at each site revealed that UVB-activated AKT phosphorylates the threonine residue at position 179 (T179) in the catalytic domain of TC45, which leads to the direct interaction of TC45 with 14-3-3σ and promotes the translocation of cytoplasmic TC45 to the keratinocyte nucleus in response to UVB irradiation. Further investigation showed that deletion of TC45 NLSII also prevented UVB-induced nuclear translocation, implying that both T179 phosphorylation and NLSII are required for efficient nuclear translocation [7]. Inhibition of TC45 nuclear translocation *via* the substitution of T179 to alanine (T179A) or the deletion of NLSII impacted the regulation of STAT3 by TC45 as evidenced by the significant increase in the level of phosphorylated STAT3 within keratinocytes following UVB irradiation. Correspondingly, loss of TC45 nuclear translocation *via* the T179A mutation inhibited apoptosis and promoted cell proliferation upon UVB exposure (Figure 1). These findings suggest that nuclear translocation of TC45 by the AKT/14-3-3σ axis is a critical protective mechanism that controls keratinocyte cell proliferation and survival in order to mitigate the propagation of UVB damaged cells.
